# Effects of dietary phosphorous supplementation on laying performance, egg quality, bone health and immune responses of laying hens challenged with *Escherichia coli* lipopolysaccharide

**DOI:** 10.1186/s40104-018-0271-z

**Published:** 2018-08-13

**Authors:** Wei Nie, Bo Wang, Jing Gao, Yuming Guo, Zhong Wang

**Affiliations:** 1 State Key Laboratory of Animal Nutrition, College of Animal Science and Technology, China Agricultural University, Beijing, 100193 People’s Republic of China; 2 Department of Animal Science, Washington State University, Pullman, Washington USA

**Keywords:** Egg quality, Immune response, Laying hens, Lipopolysaccharide, Phosphorus

## Abstract

**Background:**

Phosphorus is an essential nutrient to maintain poultry health and performance. The objective of this study was to evaluate the effect of dietary phosphorus levels on egg production, egg quality, bone health, immune responses of laying hens challenged with *Escherichia coli* lipopolysaccharide.

**Methods:**

Three hundred laying hens at 28 wk were randomly divided into 2 dietary treatments with 10 replicates of 15 birds. The wheat-soybean based diets contained either 0.12% or 0.4% non-phytate phosphorus (NPP). At 32 wk of age, all the birds of each dietary treatment were injected into the abdomen with 1.5 mg/kg body weight (BW) of either LPS or saline once a day at 24-h intervals for continuous 9 d. The performance of laying hens was evaluated for 9 d. The eggs after the fifth injection were collected to value the egg quality. Three hours after the first injection, blood was collected to measure serum metabolite and immune response associated parameters. Three hours after the fifth injection, the hens were euthanized to obtain tibia, cecal tonsils and jejunum.

**Results:**

Compared with saline-injected hens, LPS-injected hens had lower feed intake and egg production (*P* < 0.05). Eggshell thickness, strength, albumin height and Haugh unit were significantly increased in LPS-injected hens compared with saline-injected hens (*P* < 0.05). Furthermore, laying hens challenged with LPS had lower villious height/ crypt depth ration than those received saline. Serum calcium, phosphorus and SOD activities significantly decreased in the LPS-injected hens compared with the control (*P* < 0.05). LPS up-regulated expression of *IL-1β*, *IL-6* and *IL-10* in cecum, and serum concentration of MDA, IL-1β and IL-6 (*P* < 0.05), whereas 0.40% dietary non-phytate phosphorus supplementation significantly increased (*P* < 0.05) villi height/crypt depth ratio, decreased (*P* < 0.05) serum MDA and IFN-γ concentration compared with the 0.12% non-phytate phosphorus group.

**Conclusion:**

In summary, this study demonstrates that 0.40% dietary non-phytate phosphorus supplementation significantly increased calcium and phosphorus levels of eggshell, increased villi height/crypt depth ratio, decreased serum MDA and IFN-γ concentration compared with the 0.12% non-phytate phosphorus groups. The results indicate that high level of dietary non-phytate phosphorus exerts a potential effect in alleviating systemic inflammation of LPS-challenged laying hens.

## Background

In modern poultry production, birds are always confronted with various challenges, such as immunological stress, pathogen infection and environmental stress, etc. These harmful factors induce nonessential immune responses resulting in poor production, host tissue damage or clinical diseases [1–5]. Lipopolysaccharide (LPS), a component of the cell wall of gram-negative bacteria and a highly efficient proinflammatory substance, has been widely used to model bacterial infection experimentally in poultry and livestock [6–8]. Immunological challenges or oxidative stress induced by LPS or other stress factors influence the physiological and biochemical processes of animals and interferes with their normal metabolism and functions [8, 9]. In commercial practice, there is a great potential to maximize live performance of animals by regulating those stress response using dietary or pharmacologic methods [10–13].

Phosphorus (P) is an essential and expensive mineral in poultry production [14], due to its expressive participation in quality of the egg shell, the metabolic and structural function in bone and eggshell formation. P is a critical component of cell membranes, and is involved in glycolysis and other metabolic pathways [15]. In addition, farmers tend to use feed with higher level of P than recommended [16]. Previous studies had shown that the supply of dietary P is related to the regulation of immune functions [17–19]. Kegley et al. [20] found that swine lymphocyte proliferation increased in response to phytohemagglutinin (PHA), but decreased in response to pokeweed mitogen (PWM) by increasing supplemental P. Liu et al. [17] reported that phytate content and phytase activity in the poultry diets can affect the number of blood T and B cells. An increased P level in the diet of swine increased lymphocyte proliferation response, but reduced the antibody response after injection with sheep erythrocytes or ovalbumin [20]. In other species of P depletion state, immune function is impaired [21–23]. In addition, several studies of pigs have shown that dietary P and calcium (Ca) affect the bacterial microbiome activity in gastrointestinal tracts [24, 25] and have some impact on the animal immune system. Thus, the hypothesis of this study was that dietary phosphorus supplementation could attenuate the LPS-induced inflammation in laying hens. However, few studies were conducted to determine the effect of dietary phosphorus levels on production and immune response in laying hens challenged with LPS. Therefore, the present study was designed to test this hypothesis using a LPS-induced inflammatory model and evaluate the effect of dietary phosphorus levels on egg production, egg quality, bone health and immune responses in laying hens.

## Methods

### Experimental design and diets

The hens were assigned to a completely randomized study design based on a 2 × 2 factorial arrangement (Table 1). The main factors were 1) diet, basal laying hen diet with 0.12% supplemental NPP (*n* = 5) and 0.4% supplemental NPP (*n* = 5), and 2) immunological challenge, injection with LPS or saline. Three hundred Hyline Brown laying hens at the age of 28 wk were randomly divided into 2 dietary treatments with 0.12% or 0.4% non-phytate phosphorus. Each treatment consisted of 10 replicates with 15 laying hens (5 cages per replicate, 3 laying hens per cage). Dimension of the cage was measured 39 cm × 37 cm × 40 cm (width × depth × height). All hens were raised in battery cages equipped with individual feeder and water supply in an environmentally controlled room. Before the start of the experiment, all hens were fed with experimental diets for 4 wk to adapt experimental diets. The hens had free access to mash feed and water. Diets were formulated based on nonphytate phosphorus instead of a total phosphorus basis. The basal diet was a corn soybean-based ration, all nutrients except phosphorus, were formulated to meet the requirement recommended by the Chinese Chicken Feeding Standard Requirements (NY/T 33-2004) and HY-LINE VARIETY BROWN laying hen nutrient guidance. Phosphorus in the feed was analyzed using a vanadate-molybdate reagent [26]. Ingredients and nutrient composition of the experimental diets are shown in Table 1. At the age of 32 wk, 150 laying hens were intraperitoneally injected with *Escherichia coli* LPS (serotype 0111: B4, Sigma Aldrich Inc., St. Louis, MO; LPS treatment) at the dose of 1.5 mg/kg body weight (BW), another 150 laying hens were intraperitoneally injected with 0.9% (*w/v*) sterile saline at the dose of 1.5 mg/kg body weight, all hens were injected for 9 d for 5 times at 24-h intervals.

**Table 1:** Composition of the experimental laying hen diet

Items	Dietary treatments				
	LPS	–	–	+	+
	NPP,%	0.12	0.40	0.12	0.40
Ingredient, %					
Corn		61.29	60.45	61.29	60.45
Soybean meal		27.21	27.29	27.21	27.29
Soybean oil		0.97	1.21	0.97	1.21
Limestone		9.73	8.59	9.73	8.59
Dicalcium phosphate		0	1.65	0	1.65
Salt		0.30	0.30	0.30	0.30
Trace mineral premix^a^		0.20	0.20	0.20	0.20
Vitamin premix^b^		0.02	0.02	0.02	0.02
*DL*-Methionine		0.16	0.17	0.16	0.17
Choline chloride		0.12	0.12	0.12	0.12
Total		100	100	100	100
Nutrient composition, %					
ME, Mcal/kg		2.7	2.7	2.7	2.7
CP		17	17	17	17
Calcium		3.5	3.5	3.5	3.5
Nonphytate phosphorus (NPP)		0.12	0.40	0.12	0.40
Lysine		0.86	0.86	0.86	0.86
Methionine+Cystine		0.65	0.65	0.65	0.65
Tryptophan		0.21	0.21	0.21	0.21
Threonine		0.66	0.66	0.66	0.66

^a^Mineral premix provided per kilogram of diet: Mn, 100 mg; Fe, 80 mg; Zn, 75 mg; Cu, 8 mg; I, 0.35 mg; Se, 0.15 mg

^b^Vitamin premix provided per kilogram of diet: vitamin A, 12,500 IU; vitamin D_3_, 2,500 IU; vitamin E, 30 IU; vitamin K_3_, 2.65 mg; vitamin B_1_, 2 mg; vitamin B_2_, 6 mg; vitamin B_12_, 0.025 mg; biotin, 0.0325 mg; folic acid, 1.25 mg; pantothenic acid, 12 mg; niacin, 50 mg

### Blood, tissue sampling

Three hours after the first LPS or saline injection, one hen was randomly selected from each replicate, blood samples were collected from the wing vein of each hen into vacutainer tubes, the blood samples for serum were clotted at room temperature for approximately 2 h. Blood samples were then centrifuged at 3,600×*g* for 10 min. Serum were obtained and stored at − 30 °C until analysis. Serum inorganic phosphorus and calcium levels were measured using commercial kits (Nanjing Jiancheng Bioengineering Institute, Nanjing, China). The levels of serum ACTH, CORT, insulin, IL-1β, IL-6, IL-10, IFN-γ were determined by ELISA (Enzyme-Linked Immuno Sorbent Assay and RIA (radio-immune method) respectively.

Three hours after the fifth injection, one hen was randomly selected from each replicate (*n* = 5), the hens were euthanized by cervical dislocation, left tibias were collected and stored at − 30 °C until analysis, cecal tonsils were aseptically obtained and immediately snap frozen in liquid nitrogen for 2 min and stored at − 80 °C until analysis. The mid-section of the jejunum was taken and washed with saline solution, placed in 10% formaldehyde and after histological procedure stained with hematoxylin and eosin. Jejunal histology parameters were viewed and photographed using the Leica DMi8e microscope and analyzed using Cellsens Imaging software to determine the villi height and crypt depth.

### Egg production and egg quality evaluation

All eggs were collected by hand daily at 16:00 h, the egg number, the broken egg number and weight in each replicate were recorded from the first day of injection to the ninth day of the fifth injection. Average egg weight (AEW) was calculated as the mean weight of all eggs from each replicate. The feed conversion ratio (FCR) was calculated as feed consumption divided by the total egg weight (feed/egg, g/g). Mortality was recorded daily as it occurred. Feed consumption by each replicate was recorded for 9 d. Daily feed intake (DFI) was adjusted for mortalities and was calculated using the following equation: DFI = feed consumption (g)/ (hen number × time (d)). Egg production rate, average egg weight, average daily egg production, feed intake and feed/egg ratio were calculated for 9 d from the first to fifth injection.

Egg quality parameters were checked for a total of 30 eggs from each treatment. 30 eggs were collected on 1 d from each treatment to conduct egg quality measurements on ninth day. Eggs were unwashed and eggs with substantial shell contamination were not included for analysis. Egg shape index was calculated by diameter/height × 100. After breakout, albumen and the yolk were separated and weighed. Relative weights of albumen and yolk were calculated against the egg weight. The albumen height was measured using a digital micrometer head IP54 (Swiss Precision Instruments, Inc., Garden Grove, USA). Yolk color was assessed using the Roche yolk color fan. Haugh units were calculated as described by Haugh [27]. Eggshell breaking strength on the vertical axis was measured by an Instron 3360 apparatus (Instron, Canton, USA). Eggshells were weighed. The shell thickness was measured at the sharp, blunt ends and equator after removing the shell membranes using a micrometer. Eggshell color was assessed by eggshell color determination apparatus (NR, Germany). Percentage shell including the inner shell membrane was calculated from shell weight and egg weight. The eggshell (yolk) proportion (%) = eggshell (yolk) weight/egg weight × 100.

### Total RNA extraction and reverse transcription

Total RNA was extracted from cecal tonsil using TRIZOL reagent (Invitrogen), and subjected to RNase-free DNase I (Promega) digestion and purification. Two micrograms (μg) of RNA was used to synthesize the first-strand cDNA using the Superscript II First-Strand Synthesis Kit (Invitrogen). Real-time quantitative RT-PCR (RT-qPCR) were used to analyze the expression of *IL-1β*, *IL6* and *IL-10* in cecal tonsils. The primer pairs used for amplification of *IL-1β*, *IL6* and *IL-10* cDNA were listed in Table 2. RT-qPCR was performed using SYBR® Green I dye and an ABI PRISM® 7500 sequence detection system. Reactions were set up with the following thermal profile: 95 °C for 2 min, 41 cycles of 95 °C for 15 s and 60 °C for 31 s. Relative expression of mRNA was determined after normalization to *GAPDH* reference using –ΔΔCt method.

**Table 2:** Primers used for relative real-time PCR^a^

Gene	Primer sequence (5′ → 3′) ^b^	GenBank accession no.
*GAPDH*	F: GGTGGAGGAATGGCTGTCA	NM_204305
	R: CCTAGGATACACAGAGGACCAGGTT	
*IL-1β*	F: ACTGGGCATCAAGGGCTA	HM179638
	R: GGTAGA AGATGA AGCGGGTC	
*IL-6*	F: TTTATGGAGAAGACCGTGAGG	HM179640
	R: TGTGGCAGATTGGTAACAGAG	
*IL-10*	F: GCTGTCACCGCTTCTTCACCT	EF554720.1
	R: GGCTCACTTCCTCCTCCTCATC	

*GAPDH* Glyceraldehyde-3-phosphate dehydrogenase, *IL-1β* Interleukin-1 beta, *IL-6* Interleukin-6, *IL-10* Interleukin 10

^a^Primers designed by Primer Express software (Applied Biosystems, Foster City, CA)

^b^*F* Forward, *R* reverse

### Tibia strength, tibia ash, calcium and phosphorus concentration

The tibias were directly de-fleshed and the patella were removed. Tibia breaking strengths of the fresh bone were measured with a WDS-1 electric universal testing machine (Shanghai Yan Run Guang Ji Science and Technology Ltd.). Three-point bending test of metaphyseal tibia with 30 mm supporting distance and 10 mm/min test speed was performed. The tibias were air dried for 24 h at room temperature, then defatted and dried at 103 °C for 24 h, finally putted in a desiccator. Bone weight was recorded. The dried tibia was ashed at 550 °C for 18 h to determine content of ash, phosphorus and calcium. The content of phosphorus and calcium were determined by ammonium metavanadate colorimetric and EDTA titration method respectively [28].

### Statistical analysis

A completely randomized design with 2 dietary treatments and 2 levels of immunological challenge in a 2 × 2 factorial arrangement was used. To test for the effects of each treatment combination, values were subjected to ANOVA using the GLM procedure of SPSS8.0 software. Differences between means were tested using Turkey’s procedure. Results were considered statistically significant when *P* ≤ 0.05.

## Results

### Performance of laying hens

The performance results of the laying hens with LPS injection and saline injected are provided in Table 3. Average egg weight, hen-day egg production, feed intake, and the egg production rate of laying hens injected with LPS were significantly lower than those of laying hens injected with saline (*P* < 0.05). Laying hens challenged with LPS had higher broken egg rate and feed conversion ratio (feed/egg) than those received saline (*P* < 0.05). The laying hen performance of different phosphorus treatments had no significant difference between LPS and saline injected (*P* > 0.05). There was no significant interaction between LPS and NPP in the performance of laying hens (*P* > 0.05).

**Table 3:** Performance of laying hens. Data are presented as means (SD)^a^

NPP, %	LPS	Egg production rate,%	Egg weight, g	Feed/egg ratio	Feed intake, g	Hen-day egg production, g/d	Broken egg rate, %	Mortality rate,%
0.12	–	90.22	59.04	1.894	100.80	53.26	0.30	0.00
	+	62.96	56.48	2.352	83.22	35.55	2.35	1.33
0.40	–	90.22	59.29	1.858	99.32	53.50	0.32	0.00
	+	66.47	57.05	2.296	86.88	37.96	1.55	1.33
SEM		3.09	0.42	0.06	2.13	2.01	0.31	0.46
Main effect means								
NPP								
0.12		76.59	57.76	2.12	92.01	44.41	1.33	0.67
0.40		78.35	58.17	2.08	93.10	45.73	0.94	0.67
LPS								
–		90.22^b^	59.17^b^	1.88^c^	100.06^b^	53.38^b^	0.31^c^	0.00
+		64.72^c^	56.77^c^	2.33^b^	85.05^c^	36.75^c^	1.95^b^	1.33
*P*-value								
NPP		0.616	0.562	0.440	0.682	0.328	0.828	1.000
LPS		<0.001	0.003	<0.001	<0.001	<0.001	0.001	0.176
NPP × LPS		0.481	0.813	0.855	0.340	0.420	0.811	1.000

^a^Means were calculated on *n* = 5 replicates (15 laying hens per replicate) per treatment

^b-c^Within comparisons, means in a column with no common superscripts differ significantly (*P* < 0.05)

### Egg quality

Egg quality of laying hens differed among all treatment groups (Table 4). Significant interactive effects between LPS and NPP levels were observed for yolk and eggshell color of laying hens (*P* < 0.05). When birds unchallenged with LPS, ones supplemented with 0.12% NPP showed lighter eggshell color than the 0.40% NPP group (*P* < 0.05). However, the unchallenged birds fed diets with 0.4% NPP improved apparently yolk and eggshell color compared with LPS injection birds fed the diets with 0.4% NPP and unchallenged birds fed diets with 0.12% NPP.

**Table 4:** The eggshell and egg qualityof laying hens. Data are presented as means (SD)^a^

NPP,%	LPS	Shell weight/ egg weight, %	Eggshell strength, kg/cm^2^	Yolk color	Ca of eggshell, %	P of eggshell,%	Eggshell color	Eggshell thickness, mm	Haugh units	Albumen height, mm
0.12	–	11.93	3.81	6.40^b^	35.13	0.199	63.43^c^	0.322	77.82	6.17
	+	10.63	3.56	5.73^d^	35.24	0.205	64.44^bc^	0.342	82.57	6.84
0.40	–	11.54	3.88	6.77^c^	34.45	0.208	65.40^b^	0.329	77.82	6.23
	+	11.54	4.30	7.70^b^	35.08	0.201	63.38^c^	0.332	85.35	7.27
SEM		0.34	0.08	0.12	0.14	0.003	0.30	0.002	0.72	0.10
Main effect means										
NPP										
0.12		11.28	3.69	6.07^c^	35.19	0.202	63.92	0.332	80.24	6.51
0.40		11.54	4.08	7.23^b^	34.77	0.204	64.41	0.330	81.52	6.74
LPS										
–		11.74	3.85	6.58	34.79	0.204	64.41	0.326^c^	77.82^c^	6.20^c^
+		11.08	3.93	6.72	35.17	0.203	63.91	0.337^b^	83.94^b^	7.05^b^
*P*-value										
NPP		0.070	0.740	<0.001	0.261	0.808	0.445	0.695	0.294	0.206
LPS		0.187	0.082	0.490	0.381	0.935	0.401	0.016	<0.001	<0.001
NPP × LPS		0.151	0.586	<0.001	0.490	0.349	0.012	0.089	0.293	0.339

^a^Means were calculated on *n* = 5 replicates (6 eggs per replicate) per treatment

^b-d^Within comparisons, means in a column with no common superscripts differ significantly (*P* < 0.05)

Eggshell strength of the laying hens with LPS injection group was higher than those with saline group (*P* = 0.082). The colors of the egg yolk with NPP at 0.4% enhanced compared with the group at 0.12% NPP (*P* < 0.05). Eggshell thickness, haugh units and albumen height of the LPS injection laying hens were significantly improved than those of saline injected hens (*P* < 0.05). Dietary NPP level at 0.4% tended to increase the percentage of eggshell weight (*P* = 0.070) compared with the 0.12% NPP group.

### Serum biochemistry, antioxidant indicators and immune parameters

The results of the serum biochemistry and immune index of the laying hens with LPS injection and saline injected are provided in Tables 5 and 6. The contents of phosphorus and calcium in the serum of laying hens were affected by LPS injection (*P* < 0.05). The contents of serum corticosterone (CORT) and insulin of laying hens significantly increased in the LPS injection groups (*P* < 0.05). Dietary NPP level at 0.4% showed a reduced trend for laying hens serum CORT concentration compared with the 0.12% NPP group (*P* = 0.099). Compared with 0.12% NPP group, the group of 0.4% NPP had apparently lower content of serum MDA (*P* < 0.05). Significantly higher contents of serum MDA were shown in birds challenged with LPS than the group with saline. On the contrary, the challenged birds had lower serum T-SOD than those unchallenged. Furthermore, there tended to be an interactive effect between LPS and NPP for TAOC (*P* = 0.076). The LPS injection group had significantly higher serum IL-1β and IL-6 levels (*P* < 0.05). The group of 0.4% NPP significantly increased serum IFN-γ levels in laying hens compared with the group of 0.12% NPP level (*P* < 0.05). No significant interaction was observed between LPS and NPP for serum biochemistry and immune index (*P* > 0.05).

**Table 5:** Serum biochemical indices and cytokines levels. Data are presented as means (SD)^a^

NPP, %	LPS	Ca, mmol/L	P, mmol/L	ALP, IU/L	ACTH, pg/mL	CORT, nmol/L	Insulin, μIU/mL	IFNγ, pg/mL	IL-1β, ng/mL	IL-6, pg/mL
0.12	–	6.32	1.70	494.42	22.80	21.75	86.56	433.83	0.27	193.33
	+	4.30	0.95	736.08	31.42	82.35	62.09	417.76	0.53	481.61
0.40	–	6.07	1.71	642.22	24.24	15.41	71.10	500.70	0.23	304.63
	+	4.25	0.90	414.12	24.98	67.86	62.91	509.46	0.81	563.11
SEM		0.29	0.11	101.34	1.41	7.15	4.02	18.14	0.10	54.28
Main effect means										
NPP										
0.12		5.31	1.33	615.25	27.11	52.05	74.33	425.80^c^	0.39	321.45
0.40		5.16	1.30	528.17	24.61	41.63	67.00	505.08^b^	0.52	433.87
LPS										
–		6.20^b^	1.71^b^	568.32	23.52	18.58^c^	78.83^b^	467.27	0.24^c^	248.98^c^
+		4.28^c^	0.92^c^	575.10	28.20	75.10^b^	62.50^c^	463.61	0.67^b^	526.89^b^
*P*-value										
NPP		0.715	0.858	0.686	0.348	0.099	0.329	0.033	0.468	0.311
LPS		<0.001	<0.001	0.975	0.089	<0.001	0.039	0.916	0.026	0.009
NPP × LPS		0.807	0.797	0.284	0.147	0.504	0.279	0.720	0.390	0.873

^a^Means were calculated on *n* = 5 replicates (one laying hens per replicate) per treatment

^b-c^Within comparisons, means in a column with no common superscripts differ significantly (*P* < 0.05)

**Table 6:** Serum antioxidant indicators of the laying hens. Data are presented as means (SD)^a^

NPP, %	LPS	MDA, nmol/mL	TAOC, U/mL	T-SOD, U/mL	GSH-Px, U/mL
0.12	–	3.33	5.70	103.5	3173
	+	5.53	6.78	118.5	2790
0.40	–	1.98	8.95	120.1	2846
	+	3.38	3.40	107.5	2666
SEM		0.60	0.89	2.92	82
Main effect means					
NPP					
0.12		4.43^b^	6.30	111.0	2982
0.40		2.60^c^	6.18	113.8	2756
LPS					
–		2.65^c^	7.51	119.3^b^	3010
+		4.58^b^	5.09	105.5^c^	2728
*P*-value					
NPP		0.036	0.971	0.609	0.158
LPS		0.032	0.218	0.019	0.083
NPP × LPS		0.605	0.076	0.819	0.514

^a^Means were calculated on *n* = 5 replicates (one laying hens per replicate) per treatment

^b-c^Within comparisons, means in a column with no common superscripts differ significantly (*P* < 0.05)

### mRNA expression of inflammatory regulation genes in the cecal tonsil

As shown in Table 7, *IL-1β* and *IL-6* mRNA expression in the cecal tonsil of laying hens were significantly upregulated by LPS injection while downregulated *IL-10* mRNA expression (*P* < 0.05). There was no significant interaction for the *IL-1β*, *IL-6* and *IL-10* mRNA expression between LPS and NPP.

**Table 7:** The relative expression of cecal tonsil inflammatory genes of the laying hens. Data are presented as means (SD)^a^

NPP,%	LPS	*IL-1β*	*IL-6*	*IL-10*
0.12	–	1.34	4.23	3.23
	+	2.21	10.34	4.11
0.40	–	1.26	1.79	1.32
	+	4.14	14.56	3.49
SEM		0.38	1.62	0.54
Main effect means				
NPP				
0.12		1.78	7.72	3.73
0.40		2.70	8.17	2.40
LPS				
–		1.30^c^	2.84^c^	2.14
+		3.18^b^	12.45^b^	3.80
*P*-value				
NPP		0.103	0.663	0.254
LPS		0.004	0.001	0.175
NPP × LPS		0.079	0.122	0.551

^a^Means were calculated on *n* = 5 replicates (one laying hens per replicate) per treatment

^b-c^Within comparisons, means in a column with no common superscripts differ significantly (*P* < 0.05)

### Laying hens morphological development of jejunum villi

As shown in Table 8 the laying hens fed with 0.4% NPP significantly upregulated villi height/crypt depth (*P* < 0.05) when compared with the 0.12% NPP group, and the group of LPS injection reduced villi height/crypt depth apparently compared with the unchallenged laying hens (*P* < 0.05). The hens injected with LPS and supplemented with 0.12% NPP had lower villi height compared with those received saline (*P* < 0.05). The laying hens crypt depth of 0.4% NPP group was significant lower than 0.12% NPP group, however, with LPS injection, crypt depth of 0.4% NPP group was significant higher than 0.12% NPP group (*P* < 0.05). The laying hens with 0.4% dietary NPP group had higher villi height/crypt depth (*P* < 0.05) than 0.12% NPP supplementation group challenged with LPS injection. Moreover, significant interactive effects between LPS and NPP were observed for villi height, crypt depth and villi height/crypt depth (*P* < 0.05).

**Table 8:** Jejunum morphological development of laying hens. Data are presented as means (SD)^a^

NPP,%	LPS	Villi height, μm	Crypt depth, μm	Villi height / Crypt depth
0.12	–	2289^c^	409^bc^	5.74^c^
	+	2002^b^	339^cd^	6.11^c^
0.40	–	2196^bc^	312^d^	7.45^b^
	+	2231^bc^	426^b^	5.86^c^
SEM		41	14	0.16
Main effect means				
NPP				
0.12		2146	374	5.93^c^
0.40		2215	376	6.57^b^
LPS				
–		2248	366	6.50^b^
+		2117	383	5.99^c^
*P*-value				
NPP		0.396	0.858	0.018
LPS		0.117	0.387	0.048
NPP × LPS		0.046	0.001	0.002

^a^Means were calculated on *n* = 5 replicates (one laying hens per replicate) per treatment

^b-d^Within comparisons, means in a column with no common superscripts differ significantly (*P* < 0.05)

### Tibia mineral composition and breaking strength

As shown in Table 9, there was significant interaction in tibia phosphorus content of laying hens between NPP and LPS injection (*P* < 0.05). LPS-injected hens fed with diets at 0.12 and 0.4% NPP level had higher tibia P content compared with the saline-injected birds fed with at 0.12% NPP. The 0.12% NPP and unchallenged birds had lowest tibia P content compared with the other groups (*P* < 0.05). Relative to the 0.12% NPP birds, the 0.40% P group showed higher tibia Ca and P levels (*P* < 0.05). LPS injection did not affect tibia calcium and phosphorus content (*P* > 0.05).

**Table 9:** Mineral composition and breaking strength of laying hens tibia. Data are presented as means (SD)^c^

NPP,%	LPS	Tibia breaking strength, kg/cm^2^	Tibia calcium^b^, %	Tibia phosphorus^b^,%	Tibia ash^a^,%
0.12	–	171.8	33.45	10.48^e^	54.14
	+	195.8	31.46	13.45^d^	54.26
0.40	–	178.7	35.10	15.26^d^	52.79
	+	191.2	36.21	13.93^d^	55.01
SEM		7.24	0.78	0.52	0.52
Main effect means					
NPP					
0.12		183.8	32.45^e^	11.96^e^	54.20
0.40		184.9	35.65^d^	14.59^d^	54.02
LPS					
–		175.2	34.27	12.87	53.54
+		193.5	33.84	13.69	54.64
*P*-value					
NPP		0.938	0.041	0.003	0.784
LPS		0.238	0.766	0.282	0.288
NPP × LPS		0.704	0.287	0.010	0.338

^a^Results were expressed on dry-defatted weight basis of tibia

^b^Results were expressed on ash weight basis of tibia

^c^Means were calculated on *n* = 5 replicates (one laying hens per replicate) per treatment

^d,e^Within comparisons, means in a column with no common superscripts differ significantly (*P* < 0.05)

## Discussion

In the present study, LPS injection resulted in a significant reduction in egg production rate, feed intake, average egg weight in laying hens. The immunological stress reduced feed intake and daily gain of pigs following LPS injection [11, 13]. Furthermore, we also found laying hens challenged with LPS had higher broken egg rate and feed conversion ratio (feed/egg) than those received saline. However, different nonphytate phosphorus treatment showed no significant effect on these parameters. Although P is an essential nutrient to maintain health and performance, particularly in the hens. Low-Ca and low-P diets were shown to directly or indirectly depress the metabolism of nutrients in broilers [29] and the performance in layers [30–35]. Low phosphorus diets will limit the animal production performance [36]. In addition, laying hens were often confronted with various stressors, such as pathogen challenges, frequent vaccine inoculation, high temperature, which lead to immune response resulting in directing more nutrient away from body protein accretion to support the immune response and decreased growth performance [1–5]. Thus, our results showed that maintaining normal production in laying hens may require more than 0.4% NPP level when laying hens are confronted with LPS challenge.

In this current study, our results indicated that LPS-induced immunological stress not only increased the thickness and eggshell strength of laying hens, but also increased the albumen height and haugh unit of the laying hens. Egg quality was improved by LPS treatment was possibly due to lower laying rate and longer egg shell formation time. However, egg shell thickness of the birds was not affected when the daily level of phosphorus increased, which was in line with the findings of Garlich [37]. On the other hand, our results indicated that laying hens fed diets with NPP level at 0.4% had improved egg yolk color compared with 0.12% NPP treatment whether LPS injection or not. Similarly, Nie et al. [38] also demonstrated that the colors of the egg yolk significantly linearly increased as the dietary levels of nonphytate phosphorus increased. In addition, dietary NPP levels at 0.4% significantly increased eggshell color of laying hens following LPS injection compared with 0.12% NPP level. Dietary lutein carotenoids, xanthophyll and other pigment levels affect the egg yolk pigmentation, more calcium in the dietary affect the absorption of lutein, the appropriate vitamins are conducive to egg yolk pigmentation [39, 40]. Previous studies had shown that dietary P level could increase the storage of vitamin A in the mouse liver [41]. The ratio of calcium and phosphorus in the diet could affect the metabolism of calcium and vitamin A, thus affecting the eggshell and egg yolk pigmentation. Therefore, our results were indicative that dietary at 0.4% NPP could improve the colors of eggshell and egg yolk of laying hens.

In the present study, LPS injection did not affect serum ALP activity and tibia calcium and phosphorus content but decreased serum calcium and phosphorus levels, implying that LPS stress affected Ca and P metabolism. However, dietary P levels significantly increased tibia Ca and P content but had no effect on serum ALP activity and the concentration of serum calcium and phosphorus. In addition, our results also showed that LPS injection hens fed with 0.12% and 0.4% NPP level had higher tibia P content compared with the saline-injected birds fed with 0.12% NPP. Similarly, studies have shown that tibial ash content also was increased with the availability of dietary phosphorus levels [31]. Leske et al. [42] found that the deposition of calcium and phosphorus in the bone and tibia intensity linearly increased with increasing dietary phosphorus levels from 0.20% to 0.34%. Additionally, some researchers had demonstrated that acute or chronic immunological stress induced by LPS altered neuroendocrine and physiological metabolism, reduced the intake and absorption of nutrients (vitamins and trace elements), resulting in decreased concentrations of vitamins and trace elements in plasma or serum or reduced growth performance [43–45]. Thus, our results indicated that increased dietary NPP levels had a beneficial effect on maintain P homeostasis (P absorption) and bone health, especially under immunological stress.

In chickens, corticosterone (CORT) is the main active form of Glucocorticoids (GC) and also a reliable indicator of stress [8, 46]. CORT stimulated catabolism and inhibited anabolism and depressed host immunity whereas insulin has the opposite function as compared to CORT. Adrenocorticotropic hormone (ACTH) stimulation will accelerate glucocorticoid synthesis, and blood glucocorticoid levels could inhibit adrenocorticotropic hormone production, forming a feedback relationship. In this study, our results found that LPS injection significantly increased serum glucocorticoid, CORT and ACTH concentration, reduced serum insulin concentration, which indicated the LPS injection caused the immunological stress reaction of laying hens. However, dietary P level at 0.4% showed a reduced trend for serum CORT concentration compared with the 0.12% P group, suggested that supplemental higher level of P could reduce immunological stress caused by LPS injection.

Antioxidant capacity is important for body health. Poultry body can produce reactive oxygen species which would attack biological membranes and cause the formation of lipid hydroperoxide and damage of tissue. Malondialdehyde (MDA) is one of the final products of lipid oxidation, there is a strong toxic to cells, the content of MDA can reflect lipid peroxidation, and MDA was also one of the major biochemical markers to measure oxidative stress of animal [3, 47]. In this study, the higher NPP level group got significantly lower level of MDA, which may due to the appropriate NPP supplementation remit oxidative stress of laying hens. Chen et al. [48] showed that LPS challenge significantly decreased serum SOD and GSH-Px activity and significantly increased MDA content. Similarly, LPS injection significantly increased serum MDA and decreased serum superoxide dismutase (T-SOD) content in the present study, which may due to LPS injection induced free radicals production, SOD, GSH-Px had been widely consumed, and finally led to liver lipid peroxidation damage. SOD activity indirectly reflects the body’s ability to scavenge oxygen free radicals. In our study, increased dietary levels of non-phytate decreased the content of MDA serum, and reduce lipid oxidation. Wen et al. [49] showed similar result, indicating that phosphorus could reduce lipid oxidation.

P plays an important role in host physiological metabolism, and is related to the regulation of immune function [17, 19, 25, 26]. Increasing dietary phosphorus level in pig or poultry diets enhanced cellular immune response [17, 20]. P depletion or deficiency impaired immune function in some species [21, 23]. In the present study, we also evaluated the effect of dietary P levels on immune function of laying hens with LPS stress. Our results found that LPS injection successfully induced immunological via upregulating proinflammatory cytokines (IL-1β and IL-6) protein levels and mRNA expression in the serum and cecal tonsil of laying hens. Even though dietary P levels did not affect these proinflammatory cytokines production, serum IFN-γ concentration was remarkably elevated with increasing dietary phosphorus levels of laying hens, which showed that P enhanced the immune function of laying hens. Future studies will need to investigate the underlying mechanism about dietary P impact on poultry immune function.

The small intestine was the main place where gastrointestinal transited and nutrients absorbed. Yang et al. [50] found that LPS cause intestinal villi structure damage, darker crypt, villus height / crypt depth decreases. Liu et al. [51] showed that dietary arginine level and lipopolysaccharide of weaned pig intestinal villus height, crypt depth ratio and both have interaction effects. This study showed that NPP levels significantly increased villus height and crypt depth ratio, LPS significantly reduced villus height and crypt depth ratio, it reduced rate of digestion and absorption. LPS-induced immune stress blocked intestinal mucosal cell growth, the high NPP level may reduce the damage of the intestinal mucosa. Pekel et al. [52] had shown that dietary phytase supplementation could markedly increase jejunum villi height. Emami et al. [53] got similar result that negative control group resulted in lower intestinal villi height and villus height and crypt depth ratio compared with positive control group. The dietary 0.40% NPP supplementation could significantly increase the ratio of villous height to crypt depth, improve intestinal structure, enhance the digestion and absorption of nutrients, and decrease immunological stress.

## Conclusion

In summary, this study demonstrates that 0.40% dietary non-phytate phosphorus supplementation significantly increased calcium and phosphorus levels of eggshell, increased villi height/crypt depth ratio, decreased serum MDA and IFN-γ concentration compared with the 0.12% non-phytate phosphorus groups. The results indicate that high level of dietary non-phytate phosphorus exerts a potential effect in alleviating systemic inflammation of LPS-challenged laying hens.

## Abbreviations

ACTH: Adrenocorticotropic hormone
ALP: Alkaline phosphatase
BW: Body weight
CORT: Corticosterone
GC: Glucocorticoids
GIT: Gastrointestinal
IFN-γ: Interferon-gamma
IL-10: Interleukin-10
IL-1β: Interleukin-1β
IL-6: Interleukin-6
LPS: Lipopolysaccharide
MDA: Malondialdehyde
NPP: Non-phytatephosphorus
P: Phosphorus
RIA: Radio-immune method
SOD: Superoxide dismutase
